# Assessing the Evidence for e-Resources for Mental Health Self-Management: A Systematic Literature Review

**DOI:** 10.2196/mental.3708

**Published:** 2014-12-08

**Authors:** Eleni Karasouli, Ann Adams

**Affiliations:** ^1^ Institute of Digital Healthcare International Digital Laboratory University of Warwick Coventry United Kingdom; ^2^ Division of Mental Health and Wellbeing Warwick Medical School University of Warwick Coventry United Kingdom

**Keywords:** self-management, mental health, depression, bipolar disorder, eHealth, e-resources, digital technology, systematic review

## Abstract

**Background:**

In a climate which recognizes mental health as a key health improvement target, but where mental health services are increasingly over-stretched, self-management e-resources can play a potentially important role in helping to ensure people get the care and support they need. They have the potential to enable individuals to learn more about, and to exercise active involvement in, their care, and thus we see a growing interest in this area for both research and practice. However, for e-resources to become important adjuncts to clinical care, it is necessary to understand if and how they impact on patients and care outcomes.

**Objective:**

The objective of this study was to review systematically the research evidence for theory-driven and evidence-based mental health self-management e-resources; and make recommendations about strengthening the future evidence base.

**Methods:**

A comprehensive literature search in MEDLINE, EMBASE, AMED, PsycINFO, Scopus, and Cochrane Library was conducted. No limits to study design were applied. We did not restrict the types of Web-based technologies included, such as websites and mobile applications, so long as they met the study inclusion criteria. A narrative synthesis of data was performed to elaborate both the development and effectiveness of online resources.

**Results:**

In total, 2969 abstracts were identified. Of those, 8 papers met the inclusion criteria. Only one randomized controlled trial was identified. The e-resources were aimed at self-management of bipolar disorder, depression, or general mental health problems. Some of the e-resources were intended to be used as prevention aids, whereas others were recovery orientated.

**Conclusions:**

Mental health self-management e-resources have the potential to be widely effective, but our review shows it is early days in terms of development of the evidence base for them. To build robust evidence, clear guidelines are needed on the development and reporting of e-resources, so that both developers and researchers maximize the potential of a new, but rapidly evolving area.

## Introduction

### Digital Technology and Self-Management of Mental Health

Digital technology has become part of nearly everyone’s lives, with 74% of households having access to the Internet, and the average user spending about 14 hours per week on it [1]. Health services are adapting to new initiatives and are progressively using information and communication technologies (ICT) in health care [2]. The Internet can be used as a cost-effective method for providing large-scale delivery of resources and interventions with the aim of enabling people to manage their own health better. Thus, there is a clear opportunity of using ICTs to help address global resource challenges, such as costs of service delivery, workforce issues, access to services, and continuity of care [2,3].

E-mental health refers to the use of ICTs for supporting and improving mental health, via online resources, social media, and smartphone applications. (In this paper, we use the term “e-resources” as an umbrella term covering the variety of media available to support self-management of mental health problems). There is a great potential for e-mental health to enable a move toward a social model of health by empowering patients to control, effectively manage, and ultimately exercise greater choice in matters related to their health and illness. This is in line with the UK Department of Health’s “No Health Without Mental Health” strategy [4] that sets out the need for a new relationship between mental health services and service users. In particular, the report stresses that service users should be offered an active role in shaping the support that is available to them.

E-mental health is a rapidly evolving area and has the potential to be delivered to large numbers of people worldwide. In addition, research shows that individuals prefer Internet-enabled health care for mental health problems [5,6]. Thus, self-management approaches, and interventions could find, a useful platform in eHealth, utilizing e-resources to support self-management of mental health and well-being.

There is no global consensus on what self-management is. Interventions, and especially e-resources, tend to use the term “self-management” rather vaguely, often confusing it with “self-help”, and only a few provide descriptions of what self-management actually means. The World Health Organization (WHO) describes self-management as “putting patients or service users in direct control of managing their conditions by enabling them to cope in one or more of the following areas; problem solving, goal setting, identifying triggers, and indicators of deteriorating health; and responding to these themselves before relying on clinician-led intervention” [7]. We have used the WHO definition as the basis for this review. On the other hand, self-help can be defined as a standardized psychological treatment that a participant can work through independently [8]. Self-management is an activity that helps people identify the need for clinical intervention and/or self-help in the first place, and which then guides them through a process of self-led management intervention, which may or may not involve the use of a specific self-help e-tool. Even though there are a burgeoning number of self-help e-resources [9], growth in self-management e-resources for common mental health problems does not appear to have happened at the same pace. In addition, even though some self-help e-tools may also include self-management components, most often than not these components are incorporated in the self-help intervention. And as we see a growing need and demand for self-management support across the range of mental health problems experienced both by people already receiving care services, and among those who are not, self-management tools deserve more individual attention, with the evidence base for self-management e-tools needing to be established independently and disentangled from the evidence base for self-help e-tools. For this reason, this review focuses on the evidence of e-resources for the self-management of mental health.

### Aim of the Study

Self-management e-resources have been used successfully in medical conditions. There are some emerging new tools in the area of mental health, however the evidence base is unclear. Specifically, little is known about the quality of the processes used in developing the e-resources or about the scientific evaluation of their effectiveness. This review aims to address this. Therefore, the aim of this study is to review the available literature systematically to identify theory-driven and evidence-based mental health and/or well-being self-management e-resources. Specifically, this paper will: (1) describe the evidence-based self-management e-resources, (2) describe the available published evidence about the e-resources’ development and effectiveness, (3) assess their methodological quality, and (4) recommend future directions for strengthening the evidence base underpinning self-management e-resources in mental health.

## Methods

### Search Strategy

We searched 6 bibliographic databases for relevant articles published between January 1990 and November 2013: (1) MEDLINE, (2) EMBASE, (3) AMED, (4) PsycINFO, (5) Scopus, and (6) Cochrane Library. The interest in and development of e-resources is a recent phenomenon and searching papers from 1990 onward guarantees inclusion of all possible e-resources (for example, the launch date of the first mobile application, app, was 2008). Terms (subject headings and MeSH terms) relevant to e-resources (smartphone, digital technology, telehealth, app, mobile phone, Internet, eHealth, mHealth, e-source, e-tool, online, Web, and tablet), self-management (self-management and self care), and mental health (mental illness, mental health, mental disorders, anxiety, depression, mood, well-being, personal safety, and risk) were used to search the electronic databases. The terms were adapted for the individual databases as needed. Limits to “humans” and “English” were applied. Further limits were applied to exclude papers with focus on physical illness, physical activity, weight management, and phobias (eg, spider phobia). Phobias were excluded, as there are a vast number of self-management e-resources, and so this should be examined separately. Study authors were contacted if further information was required. Hand searching of references in the included papers was also performed.

### Selection Process

Paper titles and abstracts were screened for eligibility. There were two reviewers that independently screened the first 30.1% of all eligible abstracts (50/166). There was 90% initial agreement, with disagreements resolved by consensus. Both reviewers further independently screened full text papers, reaching 83% agreement. Again, disagreements were resolved by consensus.

We included papers about e-resources aimed at users concerned with their mental health or well-being. We applied strict inclusion criteria in order to investigate self-management e-resources only. Self-help and/or therapeutic e-resources were excluded. Tools also had to be interactive for inclusion, so that e-resources that contained static information or which were simply educational were also excluded. E-resources could have the form of Web-based technologies such as websites, decision support systems, or mobile applications. There was no restriction on end user age. The focus of this review forms a rather new area of research and development. Developing and testing the effectiveness of an intervention is a lengthy process and needs to go through a number of steps before a definitive trial is possible. For this reason, we did not exclude papers based on study design (papers presenting outcome data, description of e-resources and/or e-resources concepts were eligible for inclusion).

### Data Extraction and Synthesis

The first reviewer (EK) extracted data from relevant publications using a Data Extraction Form specifically developed for this systematic review and according to the Centre for Reviews and Dissemination guidance [10]. A Quality Assessment Checklist was also developed taking into consideration publication-specific contextual, pragmatic, and methodological issues [10]. The checklist assessed both the studies and e-resources reported according to 13 criteria grouped as; clear description of purpose, appropriateness of study design, main methods, e-tool development process, and theoretical frameworks used. No publications were excluded based on quality. Both reviewers independently tested both forms. Due to the variability in study designs, a narrative synthesis of data was conducted.

## Results

### Study Selection

**Figure 1:**
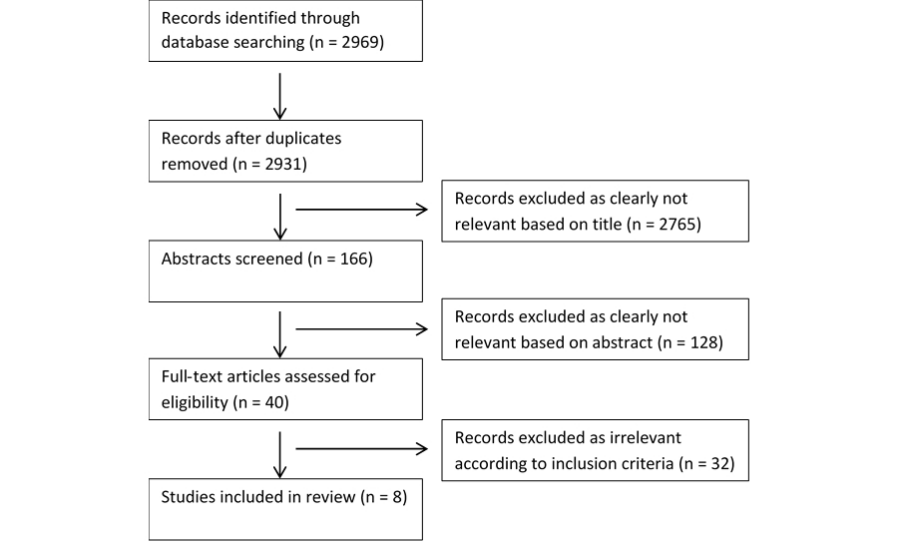
Flow diagram illustrating the study selection process.

A total of 2969 abstracts were identified from the electronic searches. There were thirty-eight of these that were removed after accounting for duplicates, leaving 2931 abstracts for further consideration. Screening the titles excluded a further 2765 records. The abstract screening process reduced the potential studies to 38. A particular abstract was based on a conference presentation; the full study was later published and picked up by our search, so the conference abstract was removed. Another abstract was excluded, as the full paper was not available (the authors of the paper were contacted, however, a copy was not sent for consideration in the review). There were four additional papers that were obtained by contacting authors of conference abstracts. In total, 40 full text papers were potentially eligible for inclusion. Of these, 32 papers were excluded, as they did not meet our inclusion criteria, identifying 8 papers suitable for the review (a sample list of excluded studies is provided, see Multimedia Appendix 1). A further screening for potentially relevant references in included studies did not reveal any additional studies. Figure 1 shows the screening process.

### Characteristics of Included Studies and e-Resources

The 8 papers identified described 2 mobile apps (Mobiletype and PHIT for Duty) [11,12], 5 interactive websites (eCHAT; SUMMIT; MyRecoveryPlan; Buddy; and Living with Bipolar) [13-17], and 1 personal digital assistant (PDA) programme (PRISM) [18]. Of the 8 included papers, successful management of bipolar disorder was described as the primary focus for 3 of the e-resources included in the review (PRISM, MyRecoveryPlan, and Living with Bipolar), depression management was the primary focus for 2 e-resources (Mobiletype and SUMMIT), and 3 papers described e-resources addressing multiple issues such as stress, anger, anxiety, and depression (PHIT for Duty), unhealthy behaviors and negative mood states (eCHAT), and general mental health problems (Buddy). In each case, the aim of the e-resource is to support the end user in achieving a reduction in the conditions and negative behaviors measured. Table 1 provides an overview of the included papers (see Multimedia Appendix 2 for a longer list).

The included papers describe e-resources addressing the needs of varied end-user populations at different stages along the care pathway; with variable degrees of integration with existing clinical service provision; and representing different degrees of progress toward generating evidence to support their efficacy and effectiveness. An e-resource targeted adolescents (Mobiletype), and 4 targeted adults (eCHAT, PHIT for Duty, SUMMIT, and Living with Bipolar). An e-resource was designed for military personnel (PHIT for Duty), 2 were designed for primary care populations (eCHAT and Mobiletype), and 2 were designed specifically for mental health service users (SUMMIT and Living with Bipolar). There were three e-resources that were intended to be used at early stages of symptoms, as prevention aids (Mobiletype, PHIT for Duty, and eCHAT), whereas, three others were recovery-orientated (SUMMIT, Living with Bipolar, and MyRecoveryPlan). There were four self-management interventions that were designed to be delivered as a stand-alone e-resource (eCHAT, Mobiletype, PHIT for Duty, and Living with Bipolar), 2 were designed to be used in conjunction with online contact either with clinicians (SUMMIT) or peer specialists (MyRecoveryPlan), 1 was designed to be accompanied by text messages (Buddy), and another one was designed as a companion to clinic-based sessions (PRISM). In terms of evidence of efficacy and effectiveness, two papers provided a general e-resource description (eCHAT and PHIT for Duty), 1 paper used mixed-methods (Buddy), and another paper described a pilot study (MyRecoveryPlan). A paper described a randomized controlled trial (RCT) protocol (Living with Bipolar), while 2 papers provided RCTs design descriptions (PRISM and SUMMIT). Only 1 paper presented a full RCT (Mobiletype).

**Table 1:** Included studies and e-resources characteristics (abridged version).

References; E-resource name	Study design	Primary outcome measures	Delivery type
Depp 2010 [18], *PRISM*	RCT (study design description)	Bipolar disorder	PDA + clinic-based sessions
Goodyear-Smith 2013 [13], *eCHAT*	General e-resource description	Unhealthy behaviors and negative mood states	Website
Kauer 2012 [11], *Mobiletype*	RCT	Depression	Mobile app
Kizakevich 2012 [12], *PHIT for Duty*	General e-resource description	Stress, depression, anger, anxiety, alcohol use, sleep quality	Mobile app
Kordy 2013 [14], *SUMMIT*	RCT (study design description)	Depression	Website; website + online chat
Simon 2011 [15], *MyRecoveryPlan*	Pilot study	Bipolar disorder	Website; website + online coaching
Treanor 2012 [16], *Buddy*	Mixed-methods	Mental health problems	Website + text messages
Todd 2012 [17], *Living with Bipolar*	RCT (protocol)	Bipolar disorder	Website

### Quality Assessment

The quality of the papers varied (see Multimedia Appendix 2). There were two papers providing only a description of e-resources that achieved a relatively high quality assessment score in the range of 4-6 out of a total possible score of 6, with a mean of 5, and standard deviation of 1.41. The 6 papers describing both evaluation studies and the prior development of e-resources achieved scores ranging from 1-13 out of a total possible score of 13, with mean of 7.7, and standard deviation of 4.55. The majority of the papers lacked information about the development process and theoretical underpinnings used to support the design of their e-resources [11,12,14-16,18]. Some papers did not provide sufficient information on how the e-resources can be accessed by users [12,14,16,18]. Finally, a few papers did not include a description of the e-resource features and components [14,16,18].

Given the lack of evidence about the efficacy and effectiveness of the mental health self-management e-resources exposed by this review, we present available evidence about the reported e-resources development processes, focussing on a number of key topics: (1) the theoretical underpinnings of the e-resources, (2) service user involvement in the development process, and (3) evaluation of acceptability and usability among the target end-user population.

### e-Resources Development Process

There were five of the publications that presented a theoretically driven approach to the development process, drawing on diverse theories related to clinical practice and work organization, education, health behavior change, and patient activation. Specifically, eCHAT builds on the self-efficacy theory of behavior change [19]. Whereas, PHIT’s approach is similar to the “subjective, objective, assessment, and plan” notes workflow model that is often used in primary care settings [20]. Living with Bipolar uses evidence-based techniques for managing mood imbalance. Theoretically, it draws on the cognitive behavioral model of mood experience [21], but also on the recovery model [22]. PRISM incorporates experience sampling [23], with aspects of an evidence-based brief psycho-educational intervention for bipolar disorder [24]. Finally, Mobiletype was developed based on the concept that self-monitoring can lead to a positive change in behavior [25-27]. Mobiletype was based on emotional self-awareness (ESA), which is hypothesized to predict depressive symptomatology [28-30].

Most of the publications did not present information on service user involvement during the development stage. Only two papers presented clear evidence of the design of the e-resources being informed by a service user perspective; those describing MyRecoveryPlan and Living with Bipolar. Service users had input into designing the content of the interventions and the Web-based formats. Early acceptability and usability testing was sometimes presented in the included publications. Papers describing both Living with Bipolar and PRISM reported previously conducted pilot studies during the development process. The PHIT paper reported that this resource was being evaluated in usability and other validation studies at the time of the review. Mobiletype’s acceptability and usability evaluation has already been published [31], and so have assessments of eCHAT [32,33].

### Use of e-Resources for Self-Management

Before going on to consider the limited available results about the efficacy and effectiveness of e-resources for mental health self-management, we present a description of the available information on the e-resources. There was marked variability in the types of content, the amount and type of user input, and method of use of the e-resources. The description of both the publications and the e-resources was also varied with respect to the details provided. To illustrate these points, we present information from the reviewed papers about the e-resources, grouped according to the mental health problems they address.

### Self-Management for Bipolar Disorder

The use of PRISM requires collaboration between users and clinicians in identifying personal mental health symptoms, illness triggers, and adaptive responses. PRISM is then personalized to prompt engagement in self-management based on real-time data. Users respond to a mood chart, and reported exacerbation of symptoms triggers the preselected self-management strategy.

Living with Bipolar is aimed at increasing access to psychological support. Users may access worksheets; record their thoughts and any symptoms; schedule activities; and create staying well plans. Living with Bipolar is expected to support users to learn about their condition, how to manage it, and increase their self-esteem. An online forum for peer support is also available.

MyRecoveryPlan may be used as a stand-alone e-tool or with the addition of online peer coaching (both in real-time and not). The e-tool uses a number of interactive sections for self-monitoring and self-management of both illness and treatment triggers. Its educational and recovery plan modules comprise of information, slide shows, and personal videos, whereas the self-monitoring modules comprise of customizable tools for tracking wellness and/or warning signs. Finally, social networking may be accessed via discussion boards, chat rooms, and peer-to-peer messaging.

### Self-Management for Depression

SUMMIT (inclusive of access to an Internet forum for peer-support) may be used as a stand-alone tool, or in combination with contact with a clinician in an online chat environment and individualized crisis management when the monitoring process signals a crisis. SUMMIT is intended for use for patients who had been treated for (at least) their third depressive episode. The primary aim of the e-resource is the promotion of self-management skills by providing continuous monitoring and supportive feedback, and allowing early detection of critical developments, as well as timely provision of clinical support.

With Mobiletype, self-monitoring data may be uploaded to general practitioners and used to guide further high-intensity interventions if needed.

### Self-Management for Mental Health in General

PHIT for Duty is aimed at those exposed to psychological trauma and showing symptoms of distress, but with subclinical findings. The e-resource is designed as a prevention aid to psychological health problems through self-monitoring and self-assessing unhealthy behaviors and negative mood states. This is the only tool in this review that builds in physiological and behavioral sensors (eg, for assessing arousal, stress reactivity, and sleep quality). It also incorporates an intelligent advisor that analyses assessments and recommends self-help interventions.

eCHAT is an e-tool designed for use as an early detection and management for lifestyle and mental health issues. It claims to focus on the whole person rather than the disease. It allows the identification of unhealthy behaviors and negative mood states so that appropriate help may be discussed with primary care clinicians. Health care professionals are able to access users’ assessment results with the aim that users play a more active role in decision-making and engagement in self-management. eCHAT may be accessed as part of a number of possible interventions, using a stepped care model.

Finally, Buddy uses a text service for Internet mood monitoring. This allows users to be able to track their moods, thoughts, and feelings. The e-tool is designed with the aim that self-reflection can help users understand the relationship between their mental health state and their daily actions. Between clinical sessions, users receive daily text messages that prompt them to record their activities and feelings. The Treanor et al [16] study does not specify if clinicians can access users’ results, or how structured the user input may be.

### Impact of e-Resources on Mental Health Self-Management

The review identified only one completed RCT. Kauer et al [11] assessed the effectiveness of Mobiletype after 2 to 4 weeks of usage. Both intervention and control groups used Mobiletype; the intervention group used an extended version of Mobiletype with additional modules on ESA, whereas the attention comparison group used an abbreviated version of the e-resource without ESA modules. The study found an indirect effect of the intervention on depressive symptoms via the mediator ESA (beta = -.610, 95% CI -5.596 to -0.003).

## Discussion

### Summary of Results

The papers included in this systematic review varied in design and purpose, ranging from descriptions of the e-resource concept and development process, through early evaluation of acceptability and usability of e-resources, to operationalized RCT protocols, and one full RCT testing the efficacy of an e-resource. The available e-resources have mixed mental health foci, with some targeting specific conditions such as bipolar disorder, while others were targeting depression, and others more general mental health issues, such as anxiety, anger, etc. Due to the limited availability of RCTs, an outcome assessment was not possible. Instead, this systematic review serves as a mapping review, presenting the available evidence about e-resources supporting self-management of mental health issues. In general, the papers lacked sufficient description of their e-resources, notably descriptions of the development process and of the built-in modules comprising the self-management intervention. The theoretical underpinnings for the approaches used were also not always clear.

### Efficacy and Effectiveness of e-Resources

The review has pointed that while e-resources addressing self-help in mental health show promising results [9], there is a dearth of studies clearly describing theoretically driven and evidence-based e-resources in mental health self-management. While new e-resources emerge daily, the evidence base supporting their use remains in its infancy. The current review found only one completed RCT [11], with a further three RCT protocols/study plan descriptions [14,17,18]. Systematic, evidence-based reporting on the development of e-resources for mental health self-management was also found to be lacking. The availability of numerous e-resources that can easily be accessed by the public without evidence of their effectiveness or of any possible harm is a worry. This is a concern across all e-health areas [34], and it necessitates the development of quality control guidelines [35].

In the absence of widely accepted guidelines for the development and evaluation of e-resources for mental health, it is advisable that the general guidelines recommended by the Medical Research Council (MRC) for complex interventions [36] are followed. There is a recent movement toward establishing guidelines for Internet intervention research that builds on the MRC’s report, but with greater relevance to the field of interest, see [37]. Both guidelines highlight the importance of testing the feasibility of interventions prior to testing their effectiveness. Testing e-resources’ usability and acceptability is especially important, as there are obvious concerns that users rarely adhere to using an e-resource for longer that just a couple of times. Qualitative research has a place in this stage so that users’ experiences in using the e-resources are explored with particular emphasis on identifying features that may or may not work for the targeted populations. Only one of the studies [16] included in this review used qualitative interviews in a mixed-methods approach, however, it is unclear at which stage of the development or evaluation process of the e-resource the study was placed, or what the study’s aims were.

### Strengths and Limitations of the Review

To our knowledge, this is the first systematic review of e-resources aimed at mental health self-management. The review presents a clear picture of the available evidence-based e-resources and highlights the need for more rigorous description and evaluation of them. Although all well-defined self-management e-resources were identified, some self-help e-resources may also incorporate some self-management components, and these would not have been identified by the review unless the self-management component was described in the study as an important element of the self-help package. No study design criteria were applied due to the low number of available studies, so outcomes cannot be summarized.

### Research and Clinical Implications

More theoretically driven and evidenced-based e-resources are needed, where the theoretical basis for developing the e-resource, together with evidence about its acceptability, usability, and effectiveness, is established in well-designed and well-reported studies. Clear guidelines to aid this process should also be implemented, so that both developers and researchers follow clear procedures.

By ensuring the rigorous evaluation of e-resources, health care professionals may then recommend the use of e-resources for self-management with confidence. They can also use self-management interventions in parallel with other health care plans, thus enabling the fulfilment of key policy visions, for example, [2,4].

### Conclusions

The area of e-health has great potential to reach wide and diverse populations, and digital technologies have huge potential for the development of effective mental health self-management e-resources. The findings of this systematic review suggest some promising developments, but they also highlight important gaps that future research should address. This is a new, but rapidly evolving, field, and while this systematic review shows plans of some good quality research currently underway, more work is needed to improve the standard of reporting of development and evaluation processes.

## Appendix

Multimedia Appendix 1 Sample list of excluded studies with reasons.

Multimedia Appendix 2 Included studies and e-resources characteristics (Table 1 full version).

## Abbreviations

app: mobile application
ESA: emotional self-awareness
GRiST: Galatean Risk and Safety Tool
ICT: information and communication technologies
MRC: Medical Research Council
PDA: personal digital assistant
RCT: randomized controlled trial
WHO: World Health Organization
