# Supplementary material for: Assessing the Evidence for e-Resources for Mental Health Self-Management: A Systematic Literature Review
Source: JMIR Ment Health. 2014 Dec 8;1(1):e3. doi: 10.2196/mental.3708 (PMC4607376; doi:10.2196/mental.3708)
Supplement: Multimedia Appendix 1 [file mental_v1i1e3_app1.pdf]

Appendix 1. Sample list of excluded studies with reasons.

**Reference**	**Reason for exclusion**
Anttila M, Koivunen M, Välimäki M (2008). Information technology-based standardized patient education in psychiatric inpatient care. J Adv Nurs, 64(2): 147-56.	Aim of resource is simply educational & used by staff
Ebert D, Tarnowski T, Gollwitzer M, Sieland B, Berking M (2013). A Transdiagnostic Internet-Based Maintenance Treatment Enhances the Stability of Outcome after Inpatient Cognitive Behavioral Therapy: A Randomized Controlled Trial. Psychother Psychosom, 82:246–256.	Aim of resource is online counselling or online therapy
Garnefski N, Kraaij V, Schroevers M (2011). Effects of a cognitive behavioral self-help program on depressed mood for people with acquired chronic physical impairments: A pilot randomized controlled trial. Patient Education and Counseling, 85: 304–307.	Static self-management information
Hirai M, Clum GA (2006). A Meta-Analytic Study of Self-Help Interventions for Anxiety Problems. Behavior Therapy, 37: 99–111.	Aim of resource is online counselling or online therapy
Robertson L, Smith M, Castle D, Tannenbaum D (2006). Using the Internet to enhance the treatment of depression. Australian Psychiatry, 14(4): 413-417.	Aim of resource is online counselling or online therapy
Simon D, Kriston L, von Wolff A, Buchholz A, Vietor C, Hecke T, Loh A, Zenker M, Weiss M, Harter M (2012). Effectiveness of a web-based, individually tailored decision aid for depression or acute low back pain: A randomized controlled trial. Patient Education and Counseling, 87: 360–368.	Aim of resource is simply educational
