# Supplementary material for: Assessing the Evidence for e-Resources for Mental Health Self-Management: A Systematic Literature Review
Source: JMIR Ment Health. 2014 Dec 8;1(1):e3. doi: 10.2196/mental.3708 (PMC4607376; doi:10.2196/mental.3708)
Supplement: Multimedia Appendix 2 [file mental_v1i1e3_app2.pdf]

Table 1. Included studies and e-resources characteristics (full version).

References; Country; E-resource Name	Study Design	Primary Outcome Measures	Study Sample	Delivery Type	Methodological Quality Rating [a]	Brief E-resource Description
Depp 2010 [18], USA, *PRISM*	RCT [b] (study design description)	Bipolar disorder	Insufficient information.	PDA [c] + clinic-based sessions	4 (out of 13)	PRISM aims to identify mood states and illness triggers, and to prompt users to engage in self-management strategies. Users respond to a mood chart with pre-selected self-management strategies appearing when exacerbation in symptoms is signalled.
Goodyear-Smith 2013 [13], New Zealand, *eCHAT*	General e-resource description	Unhealthy behaviours (risky substance use, gambling, being subject to abuse, physical inactivity) and negative mood states (depression, anxiety, anger)	Adult primary care populations.	Website	6 (out of 6)	eCHAT aims to improve health and well-being through systematic screening and intervention from modifiable lifestyle and mental health issues. A number of possible interventions can be accessed using a stepped care model.
Kauer 2012 [11], Australia, *Mobiletype*	RCT	Depression	Adolescent primary care populations.	Mobile app	11 (out of 13)	Mobiletype is a first-step intervention programme. It aims to improve emotional self-awareness by self-monitoring functioning areas such as current activities, location, companions, mood, recent stressful events, responses to stressful events, alcohol use, cannabis use, sleep quality, exercise, and diet.

Kizakevich 2012 [12], USA, *PHIT for Duty*	General e-resource description	Stress, depression, anger, anxiety, alcohol use, sleep quality.	U.S. military exposed to psychological trauma and possibly showing symptoms of distress.	Mobile app	4 (out of 6)	PHIT for Duty aims to support prevention of psychological health problems through mobile personal health assessment and self-help intervention. It integrates a suite of health assessments with an intelligent advisor that recommends, tailors, and presents self-help interventions.
Kordy 2013 [14], Germany, *SUMMIT*	RCT (study design description)	Depression	Adult mental health service users.	Website; website + online chat with clinician in real time	7 (out of 13)	SUMMIT aims to prolong symptom-free phases and to shorten symptom-loaden phases by promoting self-management by continuous monitoring and supportive feedback. Patients may use peer-support forum.
Simon 2011 [15], USA, *MyRecoveryPlan*	Pilot study	Bipolar disorder	Insufficient information.	Website; website + peer specialist online coaching	10 (out of 13)	MyRecoveryPlan aims to support users in creating and using personal recovery plans. It includes educational, recovery plan, self-monitoring, social networking and consumer activation modules. Users may create plans, use customizable tools for self-monitoring, peer-support forums, and develop calendars with clinical appointments and discussion items.
Treanor 2012 [16], UK, *Buddy*	Mixed-methods (unclear at which stage in development/ evaluation of intervention)	Mental health problems	Insufficient information.	Website + text messages	1 (out of 13)	Buddy aims to help users understand the relationship between the things they do in their daily lives and their mental state using self-reflection. Users record their daily activities and rate their mood. They also received text reminders about their clinic appointments.

Todd 2012 [17], UK, *Living with Bipolar*	RCT (protocol)	Bipolar disorder	Adult mental health service users.	Website	13 (out of 13)	The Living with Bipolar emphasis is on symptom management by giving interpersonal and practical support to allow patients to live successfully alongside their condition. Patients may use worksheets, life charts, diaries, mood checking tools, online peer forum etc.

[a] scoring range for papers that include evaluation studies: 0-13; scoring range for papers that do not include evaluation studies: 0-6.

[b] RCT: randomised control trial

[c] PDA: personal digital assistant
